# “Floating ball sign” in the diagnostic imaging of mature ovarian teratomas in children

**DOI:** 10.1007/s00383-023-05495-x

**Published:** 2023-06-16

**Authors:** Olga Szymon, Aleksandra Kiszka-Wiłkojć, Małgorzata Fryczek, Anna Taczanowska-Niemczuk, Łukasz Wyrobek, Wojciech Górecki

**Affiliations:** 1 Department of Pediatric Surgery, Jagiellonian University Medical College, University Children’s Hospital, Kraków, Poland; 2 Department of Radiology, University Children’s Hospital, Kraków, Poland

**Keywords:** Mature ovarian teratoma, Floating ball sign, Diagnostic imaging

## Abstract

The “floating ball sign” (FBS) is a rarely described visual phenomenon found in mature ovarian teratoma imaging. It is characterized by the presence of movable, spherical areas within the cystic component of a tumor. Such visualization is possible both in cross-sectional imaging and ultrasonography. To evaluate the incidence of FBS in the pediatric population with regard to patients’ age and tumor size. This is a retrospective study of pediatric patients operated on in a tertiary pediatric surgical center between January 2009 and December 2022 due to mature ovarian teratoma; the medical records were reviewed for the age at diagnosis, recurrences, tumor size, and their characteristics in preoperative imaging. Eighty-three patients (mean age 14, range 0–17) out of 91 met the inclusion criteria for the analysis. Eighty-seven operations on 90 ovaries were performed. Preoperatively 38 patients underwent CT, 13 MRI, and 39 received only the ultrasound examination. The FBS was identified in preoperative imaging diagnostics in 3 (3.3%) girls (14, 16 and 17 years of age). The average largest tumor dimension and volume were 142 mm and 1268 cc in the FBS group, and 73 mm and 252 cc in the remaining group, respectively. FBS tumors usually reach large sizes. Although the sign is rare in children, there are no scientific reports of its occurrence in the first life decade. Color flow mapping and cross-sectional imaging play a pertinent role in distinguishing this uncommon pattern from a malignant mass and enable the selection of an appropriate surgical approach.

## Introduction

Mature teratoma is the most common neoplasm of the ovary in the pediatric population [1–3]. This benign tumor derived from pluripotent germ cells may be diagnosed at any age, including infancy [4]. Its presence can be clinically silent; however, tumors greater than 5 cm pose a risk of gonadal torsion, which manifests sudden severe pain, nausea, or restlessness [5]. Large tumors may cause mass effect symptoms.

**Fig. 1:**
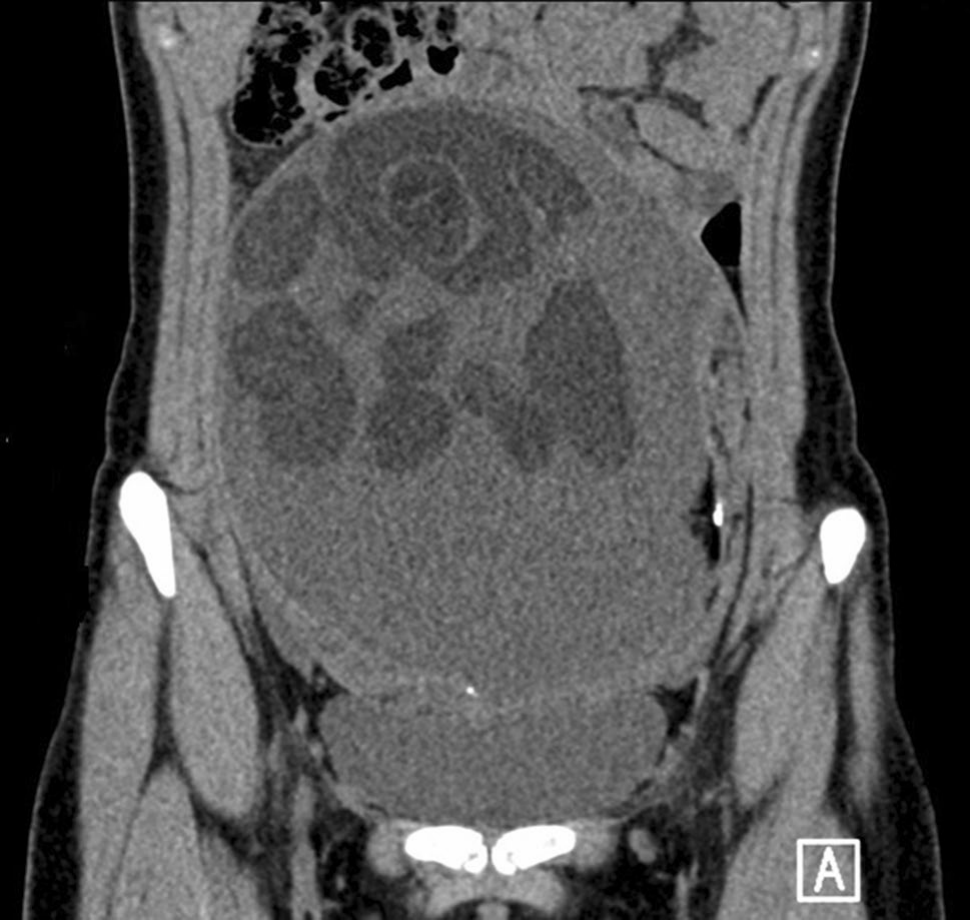
Mature ovarian teratoma with numerous floating balls visualized by computed tomography of a 16-year-old patient

**Fig. 2:**
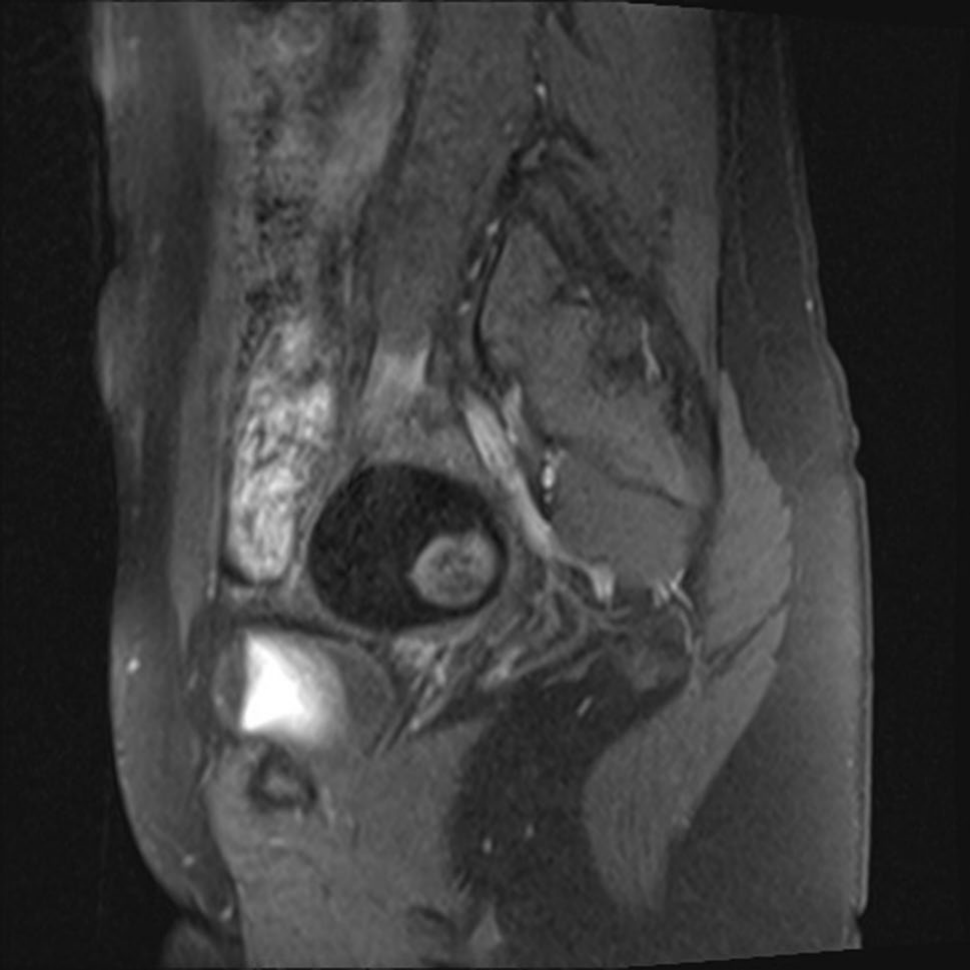
Single ball within a teratoma cyst visible in MRI scan of a 14-year-old patient

Despite the diversity of the clinical and radiological appearance, it is diagnosed preoperatively with high accuracy, which enables ovary-sparing treatment [6]. Ultrasound examination plays a major role in the diagnostic process, where the most common finding is a cystic or polycystic lesion located in the ovary with a visible sign of fatty tissue and the presence of hyperechoic calcifications. Sonographic features attributed to dermoid masses include heterogeneous echogenicity, regional diffuse bright echoes, hyperechoic lines and dots, and a visible fluid level [7, 8]. The “floating balls sign” (FBS) is a rare imaging finding characterized by the presence of hyperechoic spherules of various sizes moving freely in the hypoechoic fluid within the cystic component of a tumor (Figs. 1 and 2). This bizarre image has mostly been featured in case reports and case report series [9–13], but based on a single report by Şahin et al., while FBS may occur in 25% of cases of mature teratoma in the general population, it is rarely reported by radiologists [14].

The large size of ovarian tumors in children is an important risk factor for malignancy [1]. An unusual pattern, as well as the presence of crowded balls mimicking the nodular growth of the malignant mass, may lead surgeons to a rushed decision about the total oophorectomy. The literature describes cases of several hundred balls in one lesion [9], but even a single ball, referred to as the “Pokeball sign” [15, 16], is considered pathognomonic for mature teratoma. Since the sign has not yet been reported in children and may be unknown to many pediatric clinicians, we decided to perform a retrospective analysis of its prevalence in underage patients.

## Material and methods

The medical records of 91 pediatric patients operated on in our hospital due to ovarian teratoma between January 2009 and December 2022 were reviewed for the age at diagnosis, recurrences, metachronous diseases, tumor size, and their characteristics. Eight patients were excluded from the study due to the lack of reliable imaging documentation. The results of preoperative imaging tests of 83 patients were analyzed for the presence of the floating ball sign. All analyzed MRI examinations were performed using a standard protocol, T2-weighted images without fat saturation, T2-weighted fat-saturated images, and T1-weighted fat-saturated gradient-echo images before and after intravenous contrast administration.

CT examinations were performed before and after intravenous contrast administration.

Due to the subjective nature of the ultrasound examination, we only accepted reports meeting the following conditions: (1) the description had to include information about the size, echogenicity of structures, and location of a tumor, and (2) two or more pictures of the lesion had to be available for analysis.

Based on preoperative imaging, tumors were measured (length, width, height). Tumor volume was determined by approximating it to the volume of a sphere, where the radius was the geometric mean of half the height, width, and length of a tumor. The volume was expressed in cubic centimeters. To compare our results with previous reports, we used a test for proportions. Values of *p* < 0.05 were used to indicate significance. Institutional Review Board agreement was obtained.

## Results

For the reviewed period, 83 patients (median age 13, age range 0–17) were accepted for the analysis. Eighty-seven operations on 90 ovaries were performed. Three patients had synchronous bilateral tumors, and 4 were operated on twice due to recurrence or an asynchronous tumor in the other ovary. Mature ovarian teratoma was confirmed by a final histopathological examination in all cases.

**Fig. 3:**
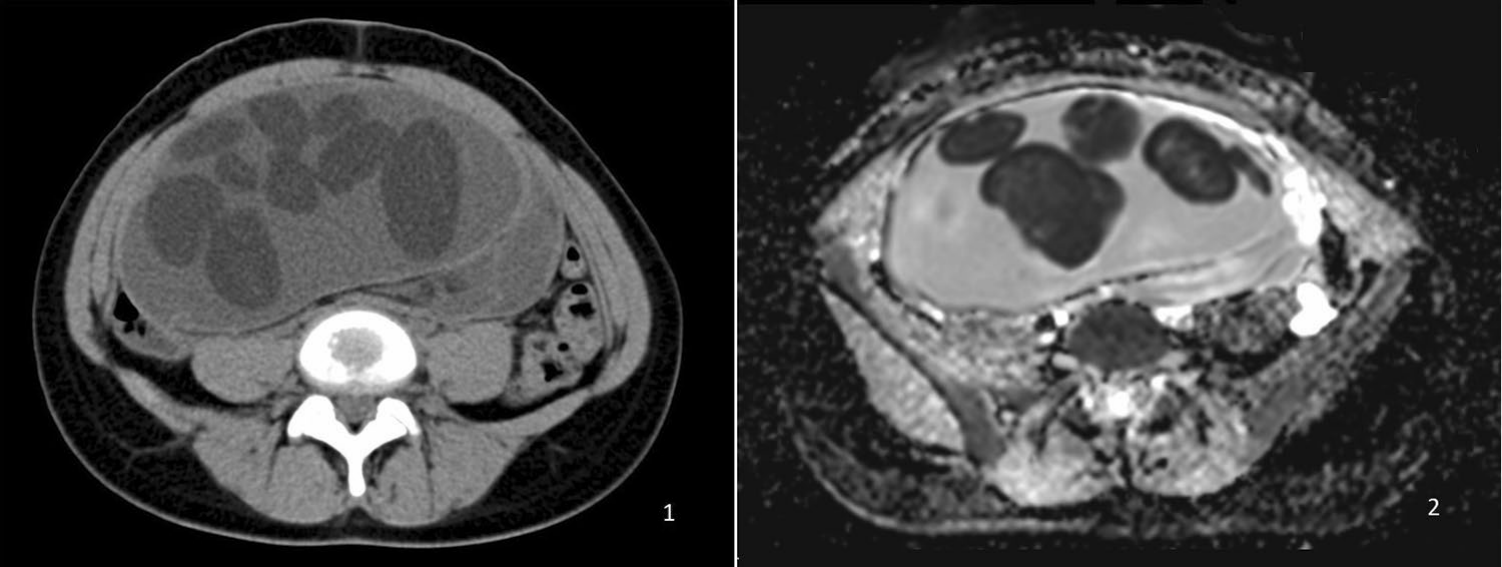
Floating balls visible in MRI (1) and CT (2) scans of a 17-year-old patient with an ovarian mature teratoma tumor

**Fig. 4:**
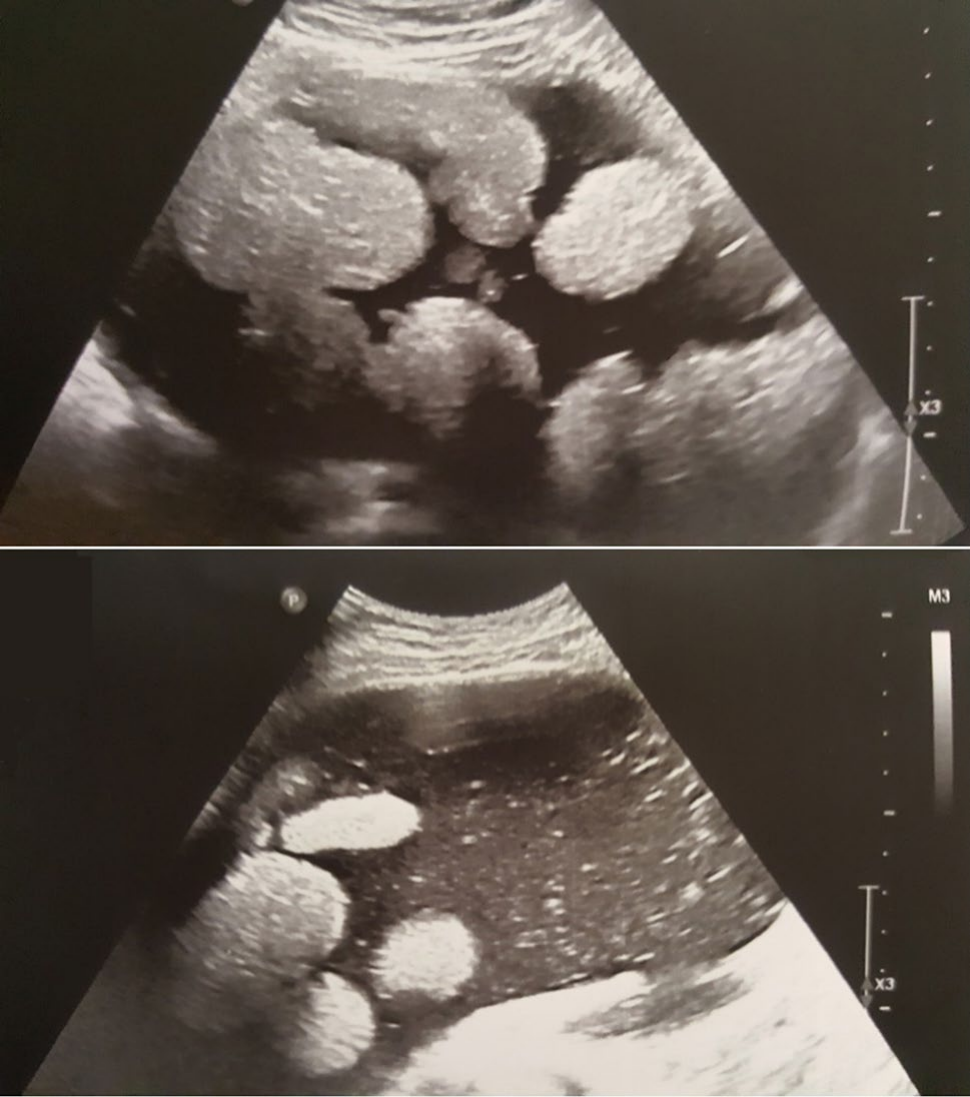
Ultrasound examination allows visualization of the movement of the balls inside the tumor cyst

**Fig. 5:**
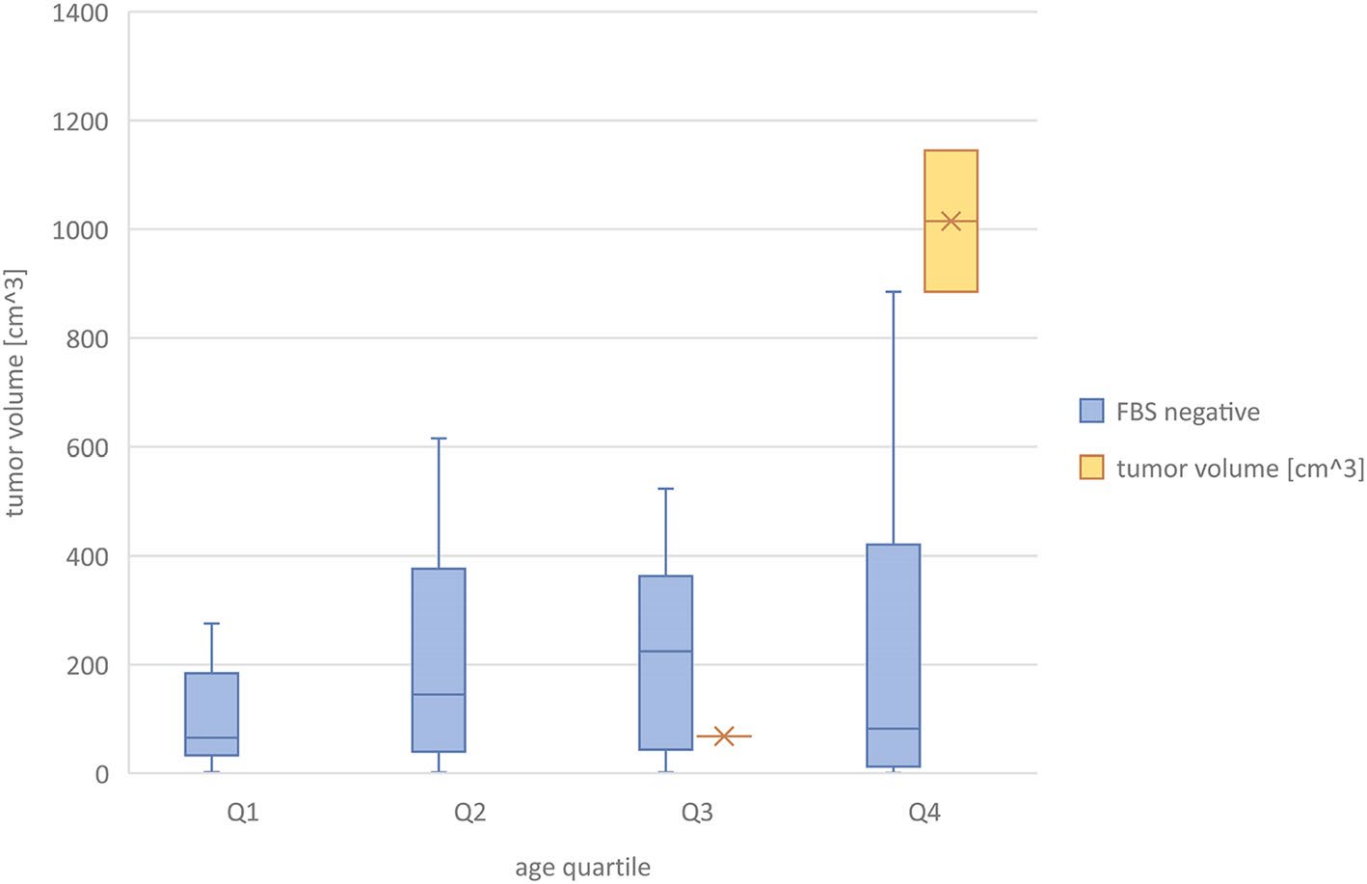
Box-and-whisker plots show tumor volume in the four groups corresponding to age quartiles. The bottom and top edges of the box indicate the 25th and 75th percentiles, respectively, and the vertical line indicates the range of data; the blue box represents the values in the FBS-negative population, while the yellow box shows FBS tumor volumes

Preoperatively, 38 girls underwent pelvic computed tomography, and 13 had magnetic resonance imaging. All patients underwent ultrasound examination; in 39 cases, the ultrasound description with multiple scans was the only imaging result analyzed. FBS was identified in preoperative diagnostic imaging in 3 (3.3%) girls (14, 16 and 17 years of age) and visualized in every preoperative imaging test performed on the patients (Table 1, Figs. 3 and 4). In those patients, the average largest tumor dimension was 142 mm, whereas in the remaining cases, it was 73 mm. The mean volume was 1268 cc for FBS and 252 cc for other teratoma tumors. Due to the low number of cases and the diversity of the pediatric population, comparative analysis by age and tumor size could not be performed. In two out of three cases, the tumor volume was the highest in the corresponding age quadrille (Fig. 5, Table 2).

**Table 1:** Patient’s baseline data and features in diagnostic imaging

Case	Age (years)	Imaging modalities	Tumor dimension (mm)	Features of the floating balls	Side
1	14	Sonography, MRI	68 × 58 × 48	One hyperechogenic ball within a cyst, size of a ball: 32 mm	Right
2	16	Sonography, CT, MRI	220 × 180 × 125	Polycystic tumor, each cyst contained multiple heterogeneous floating formations. The maximum size of a single ball 72 mm	Bilateral tumor; FBS located on the left side
3	17	Sonography, CT, MRI	152 × 137 × 105	Multiple floating balls within a single cyst. The maximum size of a single ball: 40 mm	Right

**Table 2:** Quartile characteristics

Age quartile		A	Me	SD	Ob	Kurt	Min	Max
Q1 (≤ 9 years old)	Age of the patient	5.84	7.00	2.88	− 0.51	− 1.05	0.00	9.00
	Tumor volume [cc]	153.87	65.45	234.37	3.09	10.68	1.99	1062.90
	Largest dimension [mm]	65.09	58.00	31.31	0.94	0.93	17.00	145.00
Q2 (10–13 years old)	Age of the patient	11.64	12.00	1.26	− 0.18	− 1.66	10.00	13.00
	Tumor volume [cc]	354.02	147.34	463.84	1.61	1.42	1.68	1495.40
	Largest dimension [mm]	74.79	70.00	52.78	1.23	1.22	12.00	210.00
Q3 (14–15 years old)	Age of the patient	14.32	14.00	0.48	0.84	− 1.44	14.00	15.00
	Tumor volume [cc]	276.89	219.69	302.20	1.41	0.99	1.39	966.67
	Largest dimension [mm]	83.86	83.00	40.81	0.91	1.40	19.00	193.00
Q4 (16–17 + years old)	Age of the patient	16.43	16.00	0.51	0.28	− 2.11	16.00	17.00
	Tumor volume [cc]	389.13	86.39	609.06	2.49	7.24	0.97	2591.81
	Largest dimension [mm]	80.65	73.00	52.36	0.84	0.73	13.00	220.00

*A* average, *Me* median, *SD* standard deviation, *Ob*. obliqueness, *Kurt*. kurtosis, *Min* minimum value, *Max* maximum value, *cc* cubic centimeter

## Discussion

**Fig. 6:**
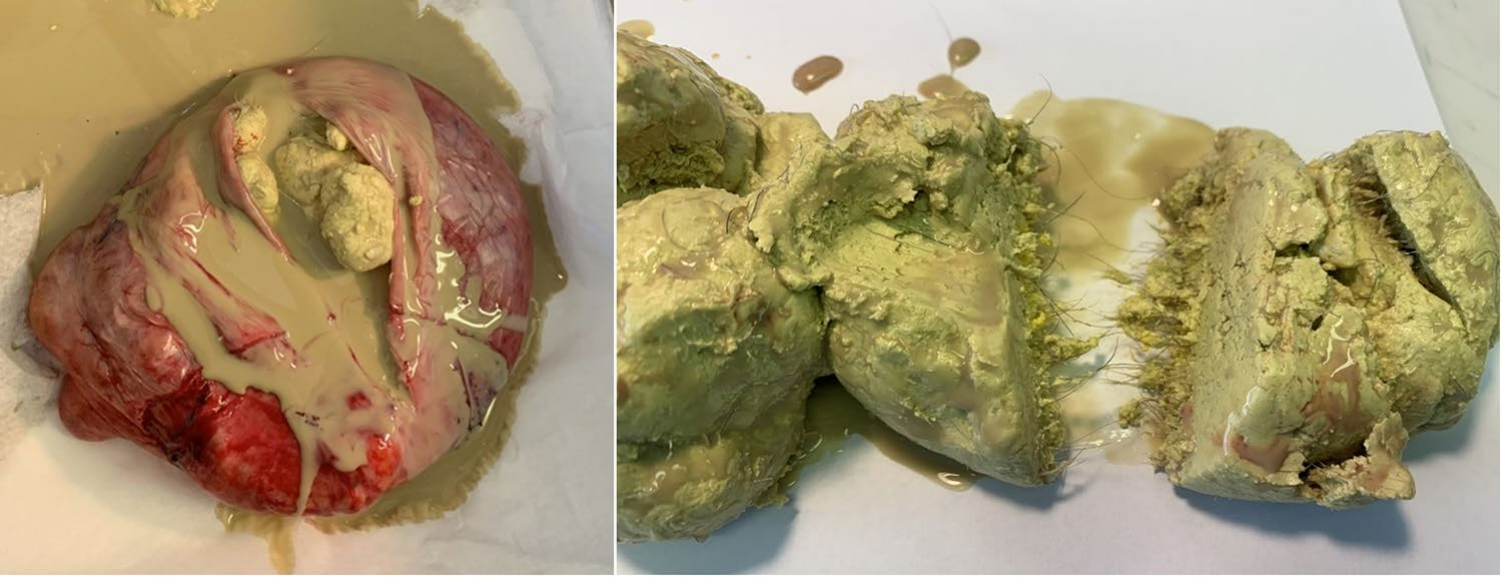
Histopathological examination revealed that “floating balls” consisted of sebum clustered around the hair shaft

The “floating ball sign” is considered pathognomonic for mature teratoma [15–19]. This unusual but unique pattern is not widely known and may cause diagnostic difficulties. The mechanism of globule formation is unknown. It is suggested that the process may be encouraged by small bowel peristalsis against the cyst wall; however, FBS teratoma was also found in the mediastinum [20] and retroperitoneum [21]. Histological findings in our three patients and the majority of reported cases suggest that balls form when sebum collects around a nidus made up of squamous or hair (Fig. 6). Such a finding, however, is not a rule. Struma ovarii, a specific monodermal subtype of mature teratoma involving thyroid tissue, may contain freely suspended beads, where the cystic spaces contain thyroid colloid, and the solid portions demonstrate thyroid follicles [22].

Şahin et al. suggest that FBS is demonstrated in 25% of ovarian teratomas in the general population; however, only patients who underwent MRI or computed tomography were included in their study. They found a significant relationship between the floating ball sign, tumor size, and the wall thickness of a tumor (*p* = 0.003 and *p* = 0.018, respectively). In 2 of their patients, mature cystic teratoma was associated with squamous cell carcinoma [14]. Presumably, the formation of the spherules requires a long time, hence the correlation between FBS and tumor size or the appearance of malignant transformation. It may also explain why our study group includes only 3 patients (3.3% of 90 operated tumors) presenting FBS in imaging diagnostics, which is statistically significantly less frequent than in the previously reported population researched by Sahin (*p* = 0.005). In two out of three cases of FBS-positive patients, the tumor volume was the highest result in the corresponding age quadrille. The recent guidelines indicate sonography as the most appropriate during the diagnostic and observation process in patients with ovarian mass and suggest the extension of diagnostics to CT or MRI in case of diagnostic uncertainty [23].

**Fig. 7:**
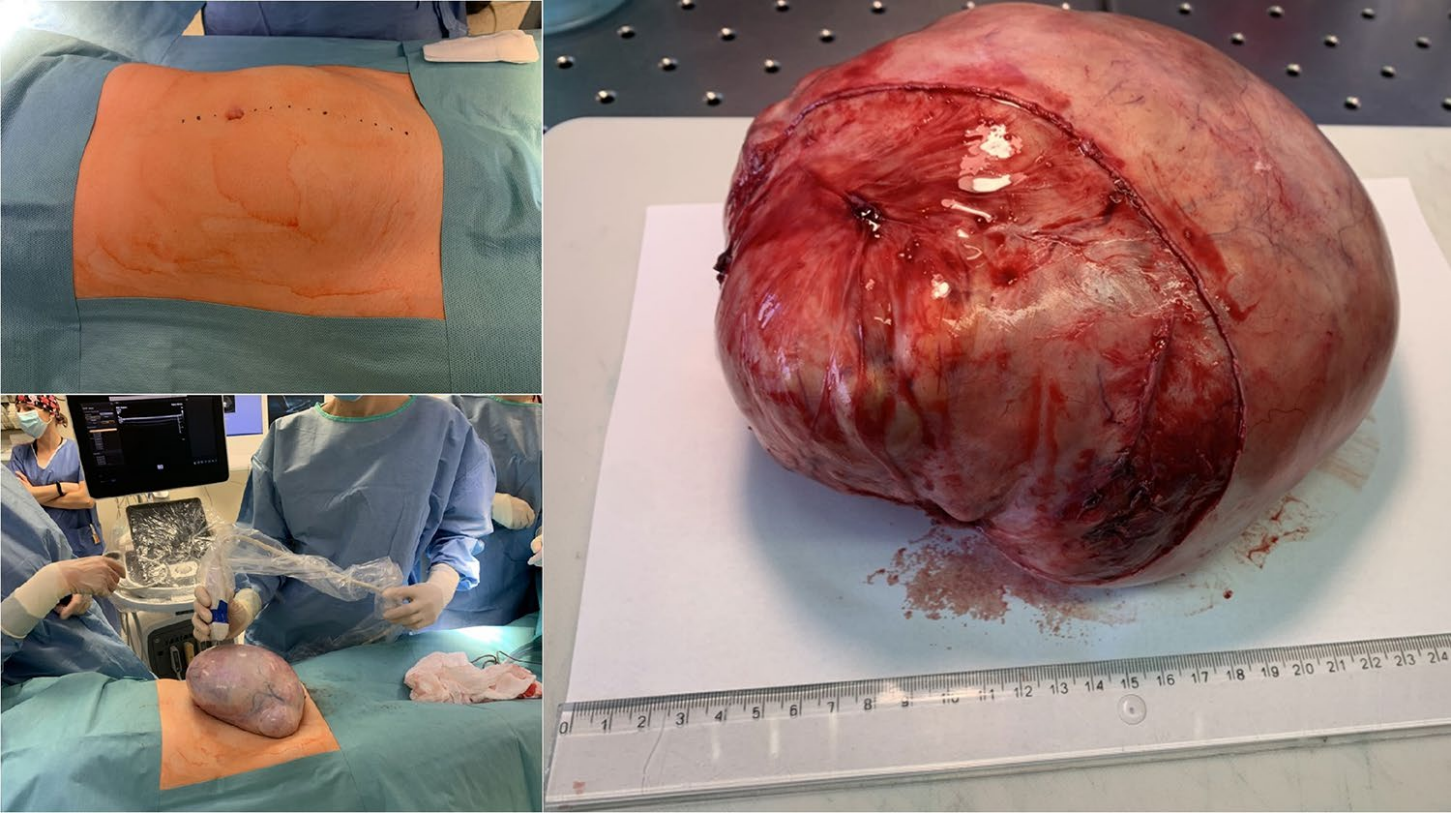
A 16-year-old patient with a floating balls tumor presenting with mass symptoms. Mature teratomas were present in both ovaries. The patient underwent a both-site ovarian-sparing tumorectomy, which allowed the majority of the ovarian cortex to be saved

The initial diagnostic test in all of our patients was the transabdominal ultrasound examination, which is less accurate than the transvaginal sonography used as standard in adult patients. Along with the variety of clinical symptoms, these limitations justify the need for cross-sectional imaging in 46 of all patients (52%). In 39 patients, the ultrasound description with multiple scans was the only imaging result analyzed. Since a significant number of our preoperative patients had only ultrasound examinations, the assessment of only cross-sectional examinations would not correspond to the actual frequency of the symptom. Aforementioned in our analysis, floating balls are visible in every type of imaging test; hence, the sign can be utilized for diagnosis with merely sonography. Nevertheless, significant tumor size and an uncommon image of the lesion may prompt the need to extend imaging and laboratory diagnostics. In one of our patients, the presence of a large polycystic tumor with floating balls resembles solid elements, and another mass in the contralateral ovary raised the fear of malignancy (Fig. 7). A similar concern can be caused by the presence of numerous crowded small balls, mimicking the nodular growth of malignant masses [9]. The important tool in distinguishing FBS teratoma is color flow mapping, which reveals the lack of vascularity within the benign cyst.

We found no other scientific reports of this sign occurring in pediatric patients. Thus, the unknown layout of a large tumor may lead surgeons to a rushed decision about the total oophorectomy. All FBS patients in our group underwent laparotomy with ovarian sparing tumorectomy. Since the malignant transformation of teratoma is mostly observed in postmenopausal patients and very rarely observed in young women [24], sparing techniques are the method of choice in ovarian teratoma management in children [6].

## Study limitations

It is a single-center retrospective analysis. The ultrasound results were assessed in the study despite their subjective nature. However, considering patients diagnosed only with MRI and CT would result in a false high prevalence of an FBS sign in the population.

## Conclusions

The floating ball sign is a rare occurrence, especially in the pediatric population, and may cause significant diagnostic and therapeutic dilemmas. All three identified in our study patients presenting FBS on preoperative imaging were teenagers, and we found no reports of the sign occurring in the first decade of life. FBS tumors usually reach large sizes. Color flow mapping and cross-sectional imaging play a pertinent role in distinguishing this uncommon pattern from a malignant mass and enable the selection of an appropriate surgical technique.

## Data Availability

The data that support the findings of this study are available on request from the corresponding author, O. Szymon.

## References

[1] Łuczak J, Bagłaj M (2017). Selecting treatment method for ovarian masses in children—24 years of experience. J Ovarian Research.

[2] Zhang M, Jiang W, Li G, Xu C (2014). Ovarian masses in children and adolescents—an analysis of 521 clinical cases. J Pediatr Adolesc Gynecol.

[3] Braungart S, Craigie RJ, Farrelly P, Losty PD, CCLG Surgeons Collaborators (2020). Ovarian tumors in children: how common are lesion recurrence and metachronous disease? A UK CCLG Surgeons Cancer Group nationwide study. J Pediatr Surg.

[4] Łuczak J, Bagłaj M (2018). Ovarian teratoma in children: a plea for collaborative clinical study. J Ovarian Res.

[5] Sosnowska-Sienkiewicz P, Mankowski P (2022). Profile of girls with adnexal torsion: single center experience. Indian Pediatr.

[6] Szymon O, Bogusz B, Taczanowska-Niemczuk A, Maślanka M, Górecki W (2021). Ovarian sparing surgery in mature ovarian teratomas in children: a 20-year single-center experience. Eur J Pediatr Surg.

[7] Patel MD, Feldstein VA, Lipson SD, Chen DC, Filly RA (1998). Cystic teratomas of the ovary: diagnostic value of sonography. Am J Roentgenol.

[8] Saleh M, Bhosale P, Menias CO (2021). Ovarian teratomas: clinical features, imaging findings and management. Abdom Radiol (NY).

[9] Tongsong T, Wanapirak C, Khunamornpong S, Sukpan K (2006). Numerous intracystic floating balls as a sonographic feature of benign cystic teratoma. J Ultrasound Med.

[10] Altinbas SK, Yalvac S, Kandemir O (2010). An unusual growth of ovarian cystic teratoma with multiple floating balls during pregnancy: a case report. J Clin Ultrasound.

[11] Otigbah C, Thompson MO, Lowe DG, Setchell M (2000). Mobile globules in benign cystic teratoma of the ovary. BJOG.

[12] Rao JR, Shah Z, Patwardhan V, Hanchate V, Thakkar H, Garg A (2002). Ovarian cystic teratoma: determined phenotypic response of keratocytes and uncommon intracystic floating balls appearance on sonography and computed tomography. J Ultrasound Med.

[13] Chang AY, Sun DC, Ohliger MA, Abuzahriyeh T, Choi HH (2020). Boba sign: a novel sign for floating balls within a mature cystic teratoma. Abdom Radiol (NY).

[14] Şahin H, Akdoğan AI, Ayaz D, Karadeniz T, Sancı M (2019). Utility of the “floating ball sign” in diagnosis of ovarian cystic teratoma. Turk J Obstet Gynecol.

[15] Giambelluca D, Caruana G, Cannella R (2021). The poké ball sign in mature ovarian cystic teratoma. Abdom Radiol (NY).

[16] Santos ARSD, de Araujo DA, Altoé A, Corrêa DG (2022). Suprasellar mature teratoma/dermoid cyst with the poké ball sign. Can J Neurol Sci.

[17] Hsiang CW, Liu WC, Huang GS, Hsu HH, Chang WC (2014). A floating ball: a pathognomonic sign of ovarian cystic teratoma. QJM.

[18] Mahomedy S, Bayat MR, Seedat M (2007). Meat balls: a pathognomonic ultrasound and computed tomography finding in mature cystic teratoma. Australas Radiol.

[19] Muramatsu Y, Moriyama N, Takayasu K, Nawano S, Yamada T (1991). CT and MR imaging of cystic ovarian teratoma with intracystic fat balls. J Comput Assist Tomogr.

[20] Hession PR, Simpson W (1996). Case report: mobile fatty globules in benign cystic teratoma of the mediastinum. Br J Radiol.

[21] Fujitoh H, Akiyosi S, Takoda S, Katsuki K, Okuda K (1998). Hepatobiliary and pancreatic imaging. Retroperitoneal mature cystic teratoma with a fat ball. J Gastroenterol Hepatol.

[22] Ye AQ, Reyes MF, Lester F (2022). Boba sign with a twist—a variant presentation of a mature cystic teratoma complicated by torsion and rupture. Clin Imaging.

[23] Luczak J, Gorecki W, Patkowski D, Baglaj M, Drosdzol-Cop A, Adamkiewicz-Drozynska E, Zaleska-Dorobisz U, Patyk M, Hirnle L (2022). Recommendations of procedures to follow in the case of ovarian lesions in girls. Ginekol Pol.

[24] Feng X, Xu L (2018). Rare case of squamous cell carcinoma arising in a recurrent ovarian mature cystic teratoma of a young woman: a case report and review of the literature. Medicine (Baltimore).

